# Differential Expression of IL-36 Family Members and IL-38 by Immune and Nonimmune Cells in Patients with Active Inflammatory Bowel Disease

**DOI:** 10.1155/2018/5140691

**Published:** 2018-12-10

**Authors:** Gabriela Fonseca-Camarillo, Janette Furuzawa-Carballeda, Emilio Iturriaga-Goyon, Jesús K. Yamamoto-Furusho

**Affiliations:** ^1^Inflammatory Bowel Disease Clinic, Department of Gastroenterology, Instituto Nacional de Ciencias Médicas y Nutrición, Salvador Zubirán, Mexico City, Mexico; ^2^Department of Immunology and Rheumatology, Instituto Nacional de Ciencias Médicas y Nutrición, Salvador Zubirán, Mexico City, Mexico; ^3^MD/PhD (PECEM) Program, Facultad de Medicina, Universidad Nacional Autonoma de México, Mexico

## Abstract

IL-1 family includes IL-38 (IL-1F10) and the subfamily of IL-36 and is the central mediators of inflammatory diseases, including pustular psoriasis, atopic dermatitis, rheumatoid arthritis, and gut inflammation. The purpose of the study was to evaluate on tissue of the patients with inflammatory bowel disease (IBD), the IL-36*α*, IL-36*β*, IL-36*γ*, IL-36Ra, and IL-38 gene and cell expression and its correlation with clinical activity.* Patients and Methods*. A cross-sectional and comparative study was performed. Seventy patients with IBD and 30 noninflamed non-IBD controls were enrolled. Gene expression was measured by RT-PCR. Protein expression was detected by double-staining immunohistochemistry.* Results*. The mRNA expression of IL-36 family members but not IL-38 was increased in colonic mucosa from patients with active ulcerative colitis versus Crohn's disease group and noninflammatory control group (*P*<0.05). However, only gene expression of IL-38 was increased in tissue from patients with inactive ulcerative colitis versus active disease and control group (*P*<0.005). Conversely, gene expression of IL-36Ra was significantly higher in colonic tissue from patients with active versus inactive ulcerative colitis and noninflamed control group (*P*<0.05). A differential protein overexpression of IL-36*α*, IL-36*β*, IL-36*γ*, IL-36Ra, and IL-38 by intestinal epithelial cells, macrophages, CD8+ T cells, and/or versus dendritic cells (pDCs) was found in patients with active inflammatory bowel disease compared with noninflamed controls*. Conclusion*. IL-38 and IL-36 family members' expression was increased by immune and nonimmune cells in patients with active inflammatory bowel disease. These cytokines and IL-36Ra might represent novel therapeutic targets in patients with gut inflammation.

## 1. Introduction

Inflammatory Bowel Disease (IBD) is characterized by an imbalance between innate and adaptive immunity leading to the stimulation of T helper responses with preponderance of proinflammatory cytokines [1]. An increase production of proinflammatory cytokines such as IL-1*β* and TNF-*α* has been involved in the development of chronic inflammation of the gut, founded on the recent knowledge about the role of cytokine-driven pathways in intestinal immunity and it is based on animal models of acute and chronic intestinal injury and inflammation [2]

The IL-1 family (IL-1F) includes IL-38 (IL-1F10) and the subfamily of IL-36 (IL-36Ra or IL-1F5; IL-36*α* or IL-1F6; IL-36*β* or IL-1F8; and IL-36*γ* or IL-1F9). It has been considered one of the most important key regulators in the pathophysiology of inflammatory autoimmune diseases including Crohn's disease (CD), rheumatoid arthritis, and psoriasis [3–5].

The IL-1 family overproduction but not from antagonist receptor (IL-36Ra) or IL-38 results in inflammation, in a robust immune response that acts as first line of defense against invasive pathogenic microorganisms and damage and when there is an aberrant immune response under appropriate genetic and environmental backgrounds in an autoimmune disease [6].

IL38/IL-1F10 is a protein that in humans is encoded by the* il1f10* gene [7]. IL-38 is expressed in a range of tissues, including heart, placenta, fetal liver, skin, spleen, thymus, and tonsil. IL-38 is also expressed mostly in the skin and in proliferating B cells [8]. This cytokine participates in a network of IL-1 family members to regulate adapted and innate immune responses by the inhibition of the production of T cell cytokines (IL-17 and IL-22). IL-38 also inhibits the production of IL-8 induced by IL-36*γ*, thus regulating inflammatory responses [9].

In addition, other secreted protein is IL-36 receptor antagonist (IL-36Ra) also known as IL-1F5, a natural inhibitor for IL-36 activity. The IL-36Ra is expressed by immune cells such as monocytes, B cells, dendritic cells/Langerhans cells, keratinocytes, and gastric parietal cells [10].

Veerdonk et al. showed that IL-38 binds to the IL-36 receptor (IL-36R) and has similar biological effects to IL-36Ra on immune cells [10]. According to its activity, low concentrations of IL-38 may have an anti-inflammatory function blocking IL-36R and IL-1R pathways, which suppressed IL-22 and IL-17 synthesis and secretion. Strikingly, IL-38 shares high sequence homology with IL-1Ra (41%) and IL-36Ra (43%) [11]

The distinct expression of IL-36*α*, IL-36*β*, and IL-36*γ*, their antagonist (IL-36Ra), and IL-38 in autoimmune disease has been shown. They showed an increased expression of IL-36*α* and IL-38 only in patients with CD but did not show the expression of IL-36 family by producing effector immune cells regarding clinical activity in patients with ulcerative colitis (UC) [5].

Nonetheless, little is known about the presence of IL-38, IL-36Ra and IL-36*α*, IL-36*β*, and IL-36*γ* producing intestinal effector immune cells (T cells, plasmacytoid dendritic cells, and monocytes) and nonimmune cells in Mexican Mestizo patients with IBD.

The purpose of the study was to evaluate on intestinal tissue IL-36*α*-, IL-36*β*-, IL-36*γ*-, IL-36Ra-, and IL-38 producing cells as well as gene expression in immune (cytotoxic T cells, macrophages and plasmacytoid dendritic cells) and nonimmune cells from patients with IBD compared with noninflamed controls.

## 2. Materials and Methods

### 2.1. Study Subjects

For the cross-sectional and comparative study, a total of 70 patients with IBD were recruited. Patients were categorized into the following groups: 30 active UC (aUC); 20 inactive UC (iUC); 10 active CD (aCD); and 10 inactive CD (iCD) patients. All patients were included between January 2014 and May 2015 from the Inflammatory Bowel Disease Clinic at the Instituto Nacional de Ciencias Médicas y Nutrición Salvador Zubirán (a tertiary referral center in Mexico City, Mexico). The UC and CD diagnosis was done by the correlation of clinical, endoscopic, and histopathological findings. Colonic samples were obtained from IBD patients and noninflamed controls after a signed informed consent. Patients' clinical records were reviewed, and personal interviews were done, and the following information were collected for all IBD patients: age, sex, treatment (mesalazine, azathioprine, prednisone, mercaptopurine, etc.), and the presence of extraintestinal manifestations (present or absent). The disease severity was evaluated in colon biopsy by Mayo score disease activity index for UC [12] and Harvey-Bradshaw Index for CD patients [13]. All colonoscopies were performed for cancer surveillance. Exclusion criteria included patients with indeterminate colitis, postradiation, infectious colitis, and other types of colitis.

The control group consisted of 30 noninflamed colonic biopsies (without endoscopic and histological evidence of any type of colitis, neoplasia, inflammatory disease, or other documented diseases). All participants underwent colonoscopy due to screening of polyps or the study of weight loss, for evaluation of anemia. Controls were matched by age and sex with IBD patients.

### 2.2. Tissue Samples

#### 2.2.1. Sample Processing and Gene Expression Analysis

To study gene and* in situ* expression we followed the methods of Fonseca-Camarillo G et al. 2015 [14]. The colonic biopsies were taken by colonoscopy and immediately submerged in RNA later solution (Ambion, Austin, TX, USA) for storage. Then total RNA was isolated using High Pure RNA Tissue (Roche Diagnostics, Mannheim, Germany), following the manufacturer's guidelines.

Electrophoresis of one aliquot was made for each one of the RNA products in an agarose gel at 1%; it was then visualized by staining with ethidium bromide and then it was documented using an UV transilluminator.

Two hundred nanograms of total RNA was reverse-transcribed into cDNA with random hexamer primers (Roche Diagnostics, Mannheim, Germany).

cDNA synthesis from total RNA through reverse transcription was made taking 20 *μ*L from total RNA of each of the products with the following protocol: preincubation: 25°C x 10 minutes, incubation: 55°C x 30 minutes, followed by denaturalization: 85°C x 5 minutes using a thermocycler (Perkin-Elmer).

Quantitative real-time PCR was used to measure the RNA transcription level of target genes. Expression of glyceraldehyde-3-phosphate dehydrogenase (GAPDH) gene as housekeeping was analyzed for normalization purposes.

PCR amplification was performed with a concentration of 20 ng of cDNA, 200 nM forward, reverse primer, and Taqman Master Mix (Roche Diagnostics, Mannheim, Germany Roche Diagnostics, Mannheim, Germany) in a final volume of 10 *μ*l. PCR reactions were run in a Light Cycler 480 (Roche Diagnostics, Mannheim, Germany).

The mRNA relative quantification of target genes was conducted using the LightCycler software 4.1, according to the 2-delta-delta Ct method.  Table 1 shows the details of the primer's designs and number of UPL (Universal Probe Library, Roche Diagnostics, Mannheim, Germany) used for the RT-PCR assay.

#### 2.2.2. Patient Samples for Protein Expression Analysis

A total of 10 surgical samples from patients with active severe UC refractory to conventional therapy were included for protein detection. Also, 10 patients with definitive diagnosis of active severe CD were enrolled in the study. Ten controls were obtained from noninflamed non-IBD intestinal tissue. All biopsies were obtained from whole colon in UC and right colon in CD patients. The Riley Index score was used for grading the severity of colonic inflammation [15].

### 2.3. Immunohistochemistry

To determine the IL-38, IL-36Ra, and secondary IL-36 family expressing cells, 4 *μ*m thick formalin-fixed and paraffin-embedded tissue from patients and noninflamed/non-IBD controls were placed on positively charged slides. Sections were deparaffinized in xylene and rehydrated in water. Morphometric evaluation of the immune-stained sections was performed in a blinded manner.

#### 2.3.1. Double-Staining Procedure

After deparaffinization and demasking of antigens with the immunohistochemistry (IHCh) enzyme antigen retrieval reagent (Enzo Life Sciences, Inc., Farmingdale, NY, USA), endogenous peroxidase was quenched with peroxidase block (Enzo Life Sciences, Inc.). Then nonspecific background staining was avoided with a serum-free solution which eliminates the need to match species with the link antibody (IHCh background blocker, Enzo Life Sciences, Inc.). To determine subpopulations of IL-38, IL-36Ra and CD14^+^/IL-36*α*, CD123^+^/IL-36*α*, CD14^+^/IL-36*β*, CD123^+^/IL-36*β*, CD8*α*^+^/IL-36*γ*, and CD123^+^/IL-36*γ* expressing cells, a simultaneous detection with a nonbiotin one-step detection was performed (MultiView (mouse-HRP/rabbit-AP) Enzo Life Sciences, Inc.).The procedure is a sequential double staining where the rabbit polyclonal anti-CD8*α* IgG or anti-CD123 IgG antibody (Santa Cruz Biotechnology, CA, USA)/mouse monoclonal anti- IL-36*γ* IgG (Abcam) and the mouse monoclonal anti-CD14 IgG2a antibody or anti-CD123 IgG1 antibody (Santa Cruz Biotechnology, Santa Cruz, CA, USA)/rabbit polyclonal anti-IL-38 IgG, anti-IL-36Ra,anti-IL-36*α*, and anti-IL-36*β* (Abcam) at 10 *μ*g/mL were incubated during 30 min at room temperature. Slides were washed with IHCh wash buffer (Enzo Life Sciences, Inc.) and then incubated with PolyView IHCh-HRP reagent for mouse antibody and PolyView IHCh-AP reagent for rabbit antibody for 20 min. Finally, antigens were visualized using horseradish peroxidase (HRP)/3, 3′-diaminobenzidine (DAB) for 10 min and the second antigen with alkaline phosphatase (AP)/Permanent Red for 5 min. Tissues were counterstained with the nonalcoholic Mayer's hematoxylin (DAKO) and mounted in aqueous mounting medium (DAKO). Negative control staining was performed with the universal negative control (a reagent mixture of purified goat, rabbit, and mouse immunoglobulins) which was tittered to work with polymer-based secondary systems (IHCh universal negative control reagent, Enzo Life Sciences, Inc.) The reactive blank was incubated with phosphate buffer saline-and IHCh background blocker instead of the primary antibody. Both controls excluded nonspecific staining and endogenous enzymatic activities. IL-38, IL-36Ra, and IL-36 producing cells were reported as the single and double positive staining cells in at least two fields (×320). Histological analysis was performed by the program Image Pro Plus version 5.1.1.

### 2.4. Ethical Considerations

This work was performed according to the principles expressed in the Declaration of Helsinki, 1989. The study was carried out with approval by the ethical committee in our institution and a written informed consent was obtained from all patients recruited prior to their inclusion in the study.

### 2.5. Statistical Analysis

Statistical analysis was performed using the SPSS 19 program by the Kruskal-Wallis One-Way Analysis of Variance on Ranks Data expressed as the median, range, and mean ± SE. A* P* value ≤ 0.05 was considered as significant.

## 3. Results

### 3.1. Demographic and Clinical Characteristics

A total of 100 individuals were recruited for the study. Patients were categorized in 4 groups: (1) active UC (n=30); (2) remission UC (n=20); (3) active CD (n=10); (4) remission CD (n=10). A fifth group consisted of 30 noninflamed/non-IBD control groups. All demographic and clinical characteristics from patients with IBD and noninflamed controls are depicted in Table 2.

### 3.2. *In Situ* Expression of IL-36RA, IL-38, and IL-36 Family in Inflammatory Bowel Disease

#### 3.2.1. IL-36Ra and IL-38

**Figure 1:**
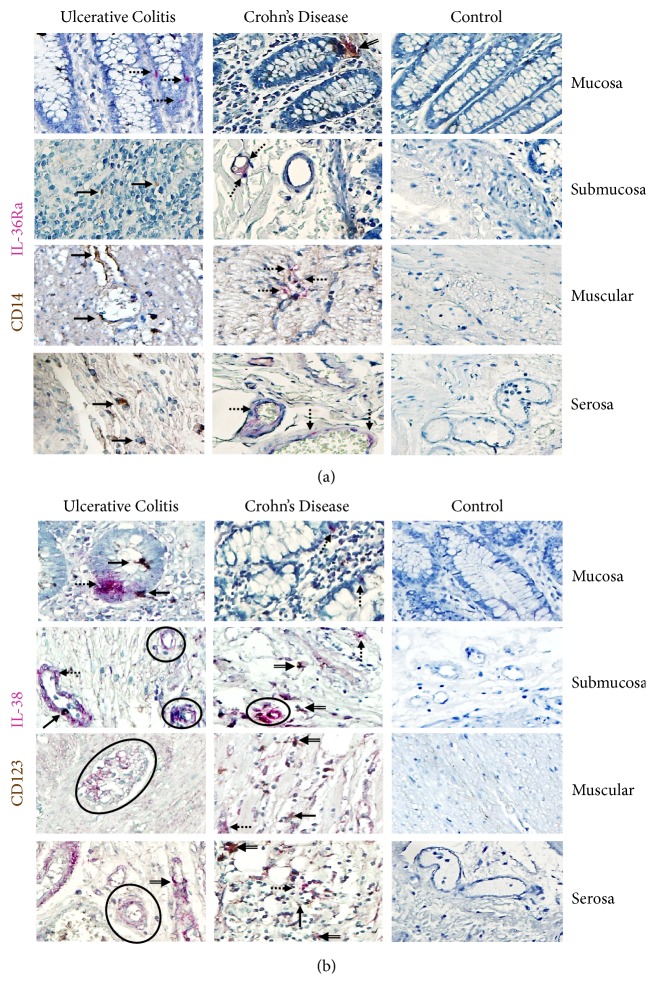
**CD14** ^**+**^ **/IL-36Ra- and CD123+/IL-38 expressing cells in intestinal tissue from IBD patients**. CD14^+^/IL-36Ra- and (b) CD123^+^/IL-38 expressing cells. Representative immunoperoxidase in tissue from active ulcerative colitis patients (n=10;* left panel*), active Crohn's disease patients (n=10;* middle panel*), and noninflamed colonic tissue (n=10;* right panel*). Photomicrographs represent mucosa, submucosa, muscular, and serosa. Dotted arrows depict the cytokine expression, solid arrows show expression of cell-surface marker (leukocytes), double arrows indicate CD14^+^ or CD123^+^ (in brown)/IL-36Ra or IL-38 (in pink) double positive cells (burgundy), and circles highlight the positive cells from perivascular inflammatory infiltrates. Original magnification was X320.

Histochemical analysis showed that the intestinal tissue from IBD patients had higher expression of IL-36Ra and IL-38 throughout the mucosa, submucosa, muscular, and serosa layers when compared with noninflamed control group. IL-36Ra producing cells were by mucosal epithelial cells and cell from submucosa and perivascular mononuclear cells. CD14^+^/IL-36Ra^+^ double positive cells from intestinal tissue from noninflamed control tissue were practically undetectable versus aUC and CD (Figure 1(a)).

The IL-38 expression in tissue from patients with UC and CD was mostly by epithelial and parenchyma cells. Nevertheless, there were some perivascular inflammatory CD123^−^ cells that expressed this cytokine. In addition, there was a small subpopulation of CD123^+^/IL-38 producing cells distributed along serosa, muscular, submucosa, and mucosa from IBD patients. The IL-38 expressing cells were plentiful in serosa, muscular, and submucosa from active UC compared to active CD and noninflamed control tissue (Figure 1(b)). The protein expression of IL-38 was scarce in mucosa from IBD patients.

#### 3.2.2. IL-36*α*

The most important IL-36*α* production was detected in nonimmune cells including gut epithelial and parenchyma cells.

Immunohistochemical double staining revealed that both CD14+ macrophages and CD123+ plasmacytoid dendritic cells expressed IL-36*α*.

**Figure 2:**
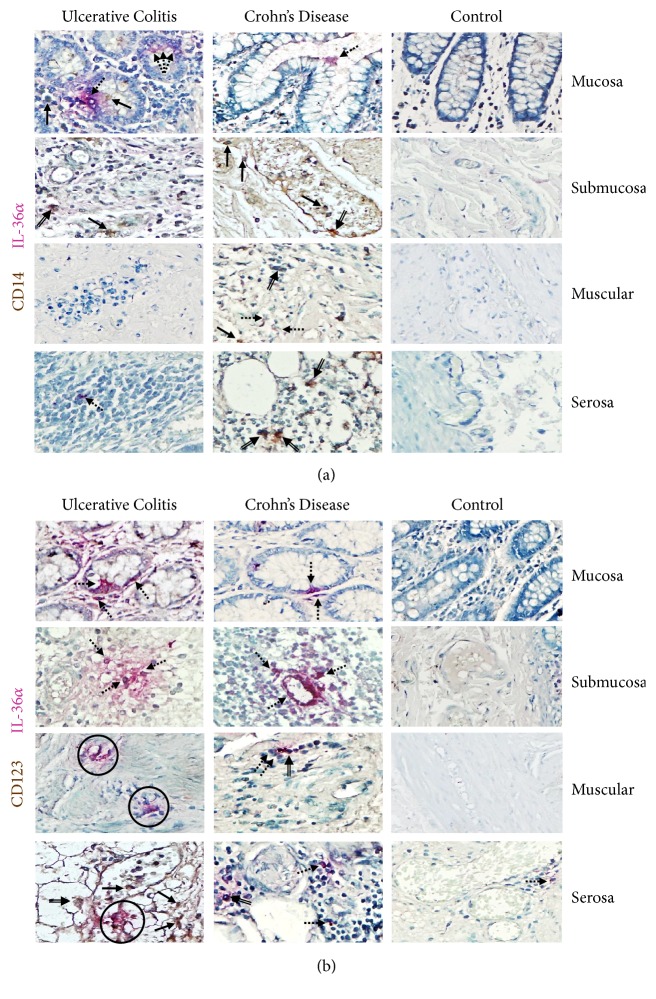
**IL-36** **α** **expression in immune and nonimmune intestinal cells from IBD patients**. (a) CD14^+^/IL-36*α* expressing cells and (b) CD123^+^/IL-36*α* pDCs. Representative immunoperoxidase in tissue from active UC patients (n=10;* left panel*), active CD patients (n=10;* middle panel*), and noninflamed colonic tissue (n=10;* right panel*). Photomicrographs represent mucosa, submucosa, muscular, and serosa. Dotted arrows depict the cytokine expression, solid arrows show expression of cell-surface marker (leukocytes), double arrows indicate CD14^+^ or CD123^+^ (in brown)/IL-36*α* (in pink) double positive cells (burgundy), and circles highlight the immunoreactive perivascular inflammatory infiltrates. Original magnification was X320.

CD14/IL-36*α* cells and CD123/IL-36*α* pDCs were more abundant on muscular and serosa from active UC patients compared with active CD patients and noninflamed control tissue (Figures 2(a) and 2(b)). The IL-36*α* expressing cells in active UC tissue were mainly CD14+ cells (Figure 2(a)), a smaller pDC positive subpopulation CD123+ (Figure 2(b)), some plasma cells, and lymphocytes. A similar pattern of IL-36*α* expression was observed in samples from CD patients. There was no expression of IL-36*α* on intestinal biopsies from controls. Staining for IL-36*α* protein in inflamed gut correlated with the pattern of mRNA expression

#### 3.2.3. IL-36*β*

**Figure 3:**
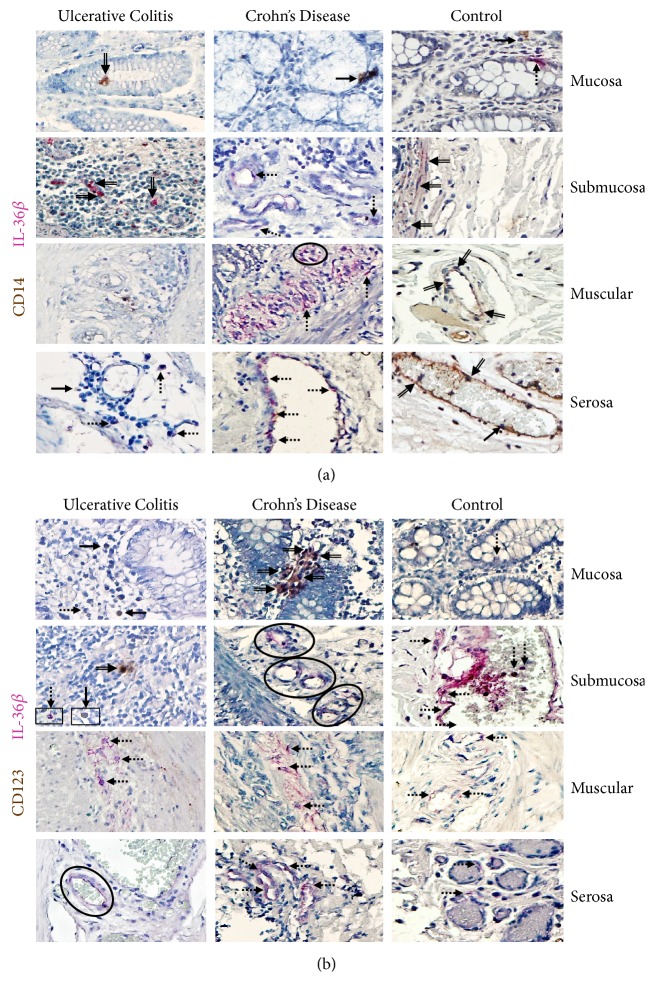
**IL-36** **β** **production in immune and nonimmune intestinal cells from IBD patients**. (a) CD14^+^/IL-36*β* expressing cells and (b) CD123^+^/IL-36*β* pDCs. Representative immunoperoxidase in inflamed tissue from active ulcerative colitis patients (n=10;* left panel*), active Crohn's disease patients (n=10;* middle panel*), and noninflamed colonic tissue (n=10;* right panel*). Photomicrographs represent mucosa, submucosa, muscular, and serosa. Dotted arrows depict the cytokine expression, solid arrows show expression of cell-surface marker (leukocytes), double arrows indicate CD14^+^ or CD123^+^ (in brown)/IL-36*β* (in pink) double positive cells (burgundy), and circles highlight the immunoreactive perivascular inflammatory infiltrates. Original magnification was X320.

CD14+/IL-36*β*+ immunoreactive macrophages were noticeably higher in active CD patients compared with active UC and noninflamed control tissue (Figures 3(a) and 3(b)). Double positive cells were localized mainly in the muscular and serosa. Furthermore, IL-36*β* producing cells, potentially lymphocytes, were increased in submucosa, muscular, serosa, and perivascular inflammatory infiltrates from active CD patients compared with noninflamed control tissue.

Biopsies from patients with active CD had a significant number of CD123^−^/IL-36 expressing cells. A conspicuous number of IL-36*β* cells were observed in serosa, muscular, and submucosa; and smaller proportion was observed in mucosa. Furthermore, in patients with active UC a considerably lower number of CD123+/IL-36*β*+ was detected in mucosa, submucosa, muscular, and serosa, although the most immunoreactive cells were CD123^−^/IL-36*β*+ immune and nonimmune cells. Tissue cells from noninflamed/non-IBD controls had a high number of IL-36*β* producing cells in serosa followed by muscular and submucosa (Figures 3(a) and 3(b)).

#### 3.2.4. IL-36*γ*

**Figure 4:**
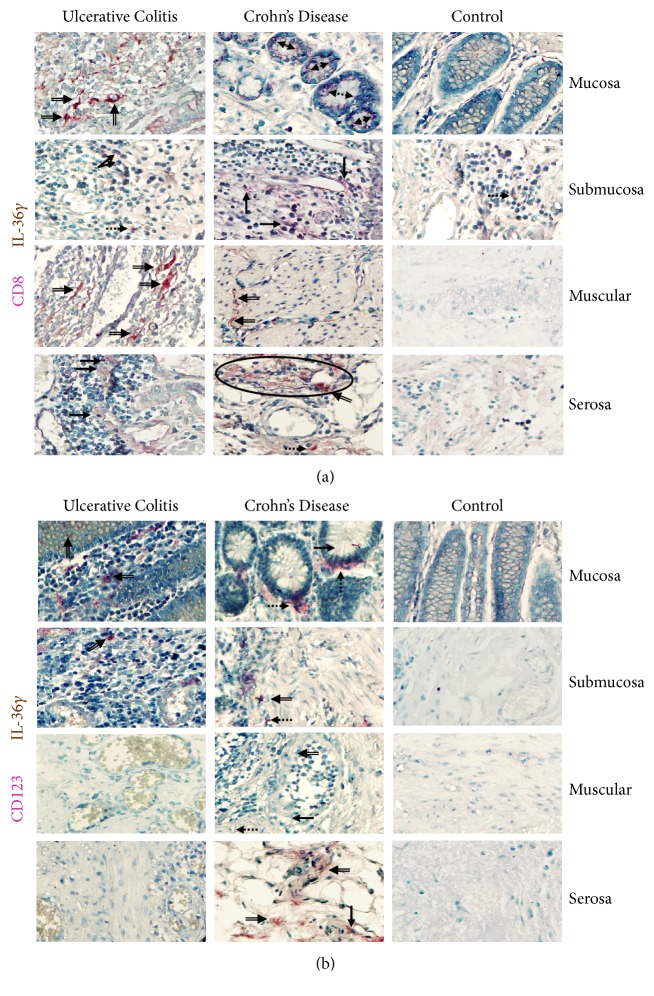
**IL-36** **γ** **production in intestinal tissue from IBD patients**. (a) CD8^+^/IL-36*γ* expressing cells. (b) CD123^+^/IL-36*γ* pDC. Representative immunoperoxidase in tissue from active ulcerative colitis patients (n=10;* left panel*), active Crohn's disease patients (n=10;* middle panel*), and noninflamed colonic tissue (n=10;* right panel*). Photomicrographs represent mucosa, submucosa, muscular, and serosa. Dotted arrows depict the cytokine expression, solid arrows show expression of cell-surface marker (leukocytes), double arrows indicate CD8*α*^+^ or CD123^+^ (in pink)/IL-36*γ* (in brown) double positive cells (burgundy), and circles highlight the immunoreactive perivascular inflammatory infiltrates. Original magnification was X320.

CD8*α*/IL-36*γ* double positive cells from patients with active UC were found in a similar proportion to the CD8*α*^−^/IL-36*γ* producing cells (potentially monocytes, lymphocytes, and plasma cells). Tissues from patients with active CD had lower number of IL-36 producing CD8*α*^+^ T lymphocytes when compared with UC. These cells infiltrate primarily the mucosa, submucosa, and muscular. In biopsies from noninflamed non-IBD patients no positive cells were determined (Figure 4(a)).

In contrast, CD123^+^/IL-36*γ*+ plasmacytoid dendritic cells were numerous on mucosa, submucosa, muscular, and serosa from active CD patients compared with active UC patients and noninflamed control tissue

Inflammatory infiltrates from CD biopsies had a significant number of IL-36*γ* expressing cells. They were distributed throughout all layers of the submucosa (and deeper layers), whereas in UC they were accentuated in the upper submucosa near the mucosal border (predominant mucosal inflammation in UC) (Figure 4(b)).

### 3.3. Gene Expression of IL-38, IL-36RA, and IL-36 Family in Colonic Mucosa from UC and CD Patients

The IL-38, IL-36Ra, and IL-36 family gene expression were detected and quantified by RT-qPCR in colonic biopsies of UC and CD patients as well as noninflamed control tissue. Bars show mean ± standard error of the mean of transcript levels from patients with GAPDH as housekeeping gene determined by 2-∆∆Ct. ^*∗*^P value <0.05 was considered as significant.

**Figure 5:**
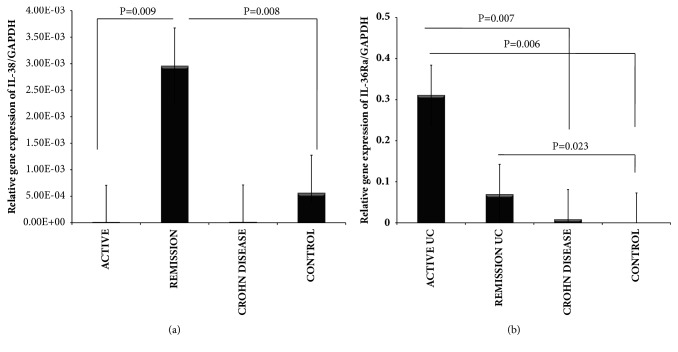
**IL-38 and IL-36Ra gene expression in IBD patients**. (a) IL-38 and (b) IL-36Ra relative gene expression quantified by RT-PCR. Bars show mean ± standard error of the mean of transcript levels in colonic mucosa from IBD patients with GAPDH as housekeeping gene determined by 2-∆∆Ct.

The gene expression of IL-38 was increased in colonic tissue from noninflamed patients with UC when compared with inflamed UC and the control group (P= 0.009 and P= 0.008, respectively). No statistically significant difference was found among patients with active UC compared with noninflamed CD (Figure 5(a)).

The gene expression of IL-36Ra was significantly higher in inflamed colonic tissue from patients with active UC when compared with inactive UC and noninflamed control group (P= 0.006 and P= 0.007). (Figure 5(b)).

**Figure 6:**
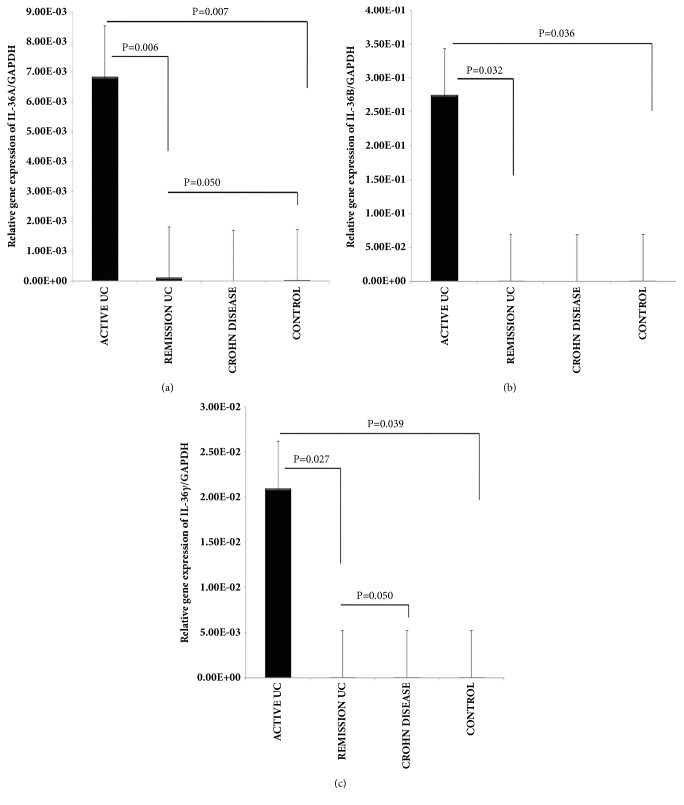
**IL-36 family relative gene expression quantified by RT-PCR**. (a) IL-36*α* gene expression. (b) IL-36ß gene expression, (c) IL-36*γ* gene expression. RT-qPCR was performed to assess mRNA levels in colonic mucosa biopsies from IBD patients, bars show means with standard error of the mean of IL-36*α*, IL-36ß and IL-36*γ* transcript levels with GAPDH as housekeeping gene determined by 2 ∆ ∆Ct, differences among groups were assessed by Kruskall Wallis test, and p values are presented in the figure.

The gene expression of IL-36*α* was significantly increased in colonic inflamed tissue of patients with active UC when compared with inactive UC and noninflamed control group, respectively (P= 0.006 and P= 0.007; Figure 6(a)). We also found a significant difference among inactive UC versus inactive CD group as shown in Figure 6(a) (P= 0.050).

IL-36*β* gene expression was higher in inflamed colonic mucosa from patients with active UC when compared with inactive UC and controls (P= 0.032 and P= 0.036; Figure 6(b))

IL-36*γ* gene expression was also significantly up-regulated in colonic mucosa from patients with active UC in comparison with inactive UC and control group (P= 0.02 and P= 0.03; Figure 6(c)). We also determined a significant difference among inactive UC versus CD group (P= 0.050; Figure 6(c)).

## 4. Discussion

To the best of our knowledge, this study demonstrated the intestinal expression of IL-38 and IL-36 family members expression by immune and nonimmune cells in patients with inflammatory bowel disease.

The most recently identified IL-36 family members are widely expressed in inflammatory, epithelial, and other nonimmune cells. These cytokines combine with the cell-surface heterodimeric receptor IL-36R (IL1RAcP/IL-1Rrp2) and activate downstream nuclear transcripts such as nuclear factor-*κ*B (NF-*κ*B) and activator protein-1 (AP-1) and MAPKs pathways like IL-1. Therefore, most molecules involved in IL-36-induced signaling, such as MMPs, antimicrobial peptides, cytokines, chemokines, and adhesion molecules, all of them mediators of inflammatory diseases [9]. IL-36 cytokine has significant* in vivo *effects on DCs and T cells in human immune responses via its role in the differentiation of inflammatory Th1 cells [16].

To identify the different cell populations of immune system we followed the methods of Fonseca-Camarillo G. et al. 2015 [14] and we detected different subpopulations such as CD8*α*^+^ T cells, CD123^+^ pDCs and CD14^+^ monocytes, and nonimmune cells including epithelial and parenchyma cells that were remarkably increased in active UC patients compared with active CD and noninflamed controls.

Previously, IL-36*α* has been reported as an IL-1 family member expressed on monocytes, macrophages, and/or dendritic cells, T cells [11], but we also showed the protein expression of IL-36*γ* by CD8 T cells and plasmacytoid dendritic cells on intestinal tissue from patients with IBD.

Russell et al. [17] recently demonstrated that expression levels of IL-36*α* are specifically elevated in the colonic mucosa of UC pediatric patients and this finding was also reported in the inflamed colonic mucosa of mice, wherein Il36r^−/−^ exhibited reduced disease severity in acute dextran sulfate sodium- (DSS-) induced model of colitis of IBD.

Kanda et al. demonstrated that IL-36*α* and IL-36*γ* contribute to gut inflammation through the induction of proinflammatory mediators such as IL-6 and CXC chemokines (CXCL1, CXCL2, and CXCL8) by human colonic subepithelial myofibroblasts [18].

Moreover, Boutet et al. reported that IL-36*α*, IL-36*γ*, and IL-38 were induced at low levels and correlated with IL-1*β*, M-CSF, and some chemokines but not with IL-17A in the colon of mice with DSS-induced colitis and in patients with CD when compared with psoriasis [5]. Besides, IL-36 and IL-38 share a common receptor, IL-36R, and exhibit dual proinflammatory effects on autoimmune diseases, particularly in psoriasis and rheumatoid arthritis. These are secreted by Langerhans cells, keratinocytes/stratified squamous epithelium, chief cells, and parietal cells [8].

Medina Contreras showed that IL-36R-deficient (Il1rl2^(-/-)^) mice exhibited defective recovery following DSS-induced damage and an important reduction in IL-22 expression, a cytokine involved in tissue repair and regeneration, particularly by colonic neutrophils and suggest the important role of IL-36/IL-36R axis in the resolution of intestinal mucosal wounds [19].

We also decided to explore the gene and protein expression of IL-38 and IL-36Ra receptor in intestinal tissue from patients with IBD. We found that IL-38 and IL-36Ra mRNA expression were increased in the tissue from active and remission IBD patients compared with noninflamed tissues.

Analysis of the whole samples showed that IL-38 mRNA levels were higher in colonic mucosa from patients in remission UC when compared with active UC. Interestingly, IL-38 gene expression was decreased in patients with active UC, and, conversely, percentage of IL-38 immunoreactive cells in active UC patients and active CD were increased compared with noninflamed tissues.

Increased IL-38 protein expression in mucosa from patients with active IBD suggests that the upregulation is a defense mechanism in the colonic epithelia in response to decreased bacterial invasion and inflammatory activity.

An increase of IL-36Ra mRNA expression was determined in active UC patients versus inactive CD patients.

Those results are relevant because previously it has been reported that the binding of IL-1Ra and IL-36Ra to their receptor reduces inflammation by blocking the binding of receptor ligands. Yuan et al. suggest the regulatory role of IL-38 by the production of fungal-induced IL-17, IL-22, and IL-36*γ*-derived IL-8 was decreased by IL-38, which may play an important anti-inflammatory role in inflammatory diseases [9].

This study described the expression of IL-36 family, IL-36Ra, and IL-38 in colonic cells and immune cells in patients with IBD.

Additional functional studies about IL-36 family and IL-38 in the gut mucosal immune response can confirm its role and support the proinflammatory role of this cytokines in patients with IBD.

There are some limitations of our study. As expected, it would be desirable to include a group of intestinal tissue from patients with remission IBD for immunohistochemistry analysis, but it is not possible since this set of patients do not need to be colectomized for treatment of the disease.

It is important to note that this study evaluated the presence of IL-36 family, IL-36Ra, and IL-38 in monocytes, CD8 T cell, and plasmacytoid dendritic cell subpopulations in IBD patients.

Summing up, current knowledge supports the concept that the pathophysiology of IBD is characterized by a robust elevation of IL-36 family members and IL-36Ra and IL-38 probably promoting agonist and/or healing activity, whose primary source is mononuclear cells and epithelial cells.

Our findings showed that clinical active IBD patients have an increased gene expression and production of IL-36Ra by macrophages. The IL-38 and IL-36Ra-related signaling pathway is poorly understood and certainly requires further studies to elucidate its role in patients with IBD.

In conclusion, our results suggest the role for IL-38 and IL-36R*α* signaling in the colonic inflammation by effector immune cells and indicate that the IL-36Ra and IL-38 pathway may represent an innovative and rational target for therapeutic treatment in patients with IBD.

## Figures and Tables

**Table 1:** Primers designs.

**Gene**	**Genebank**	**Oligonucleotides**	**Probe UPL**
*IL-36α*	NM_000572.2	CATAAATTAGAGGTCCAAAATCG AAGGGGCTGGGTCAGCTAT	#45
*IL-36ß*	NM_001558.3	GTCTTGGCTCAGACGCTCAT TGCTTCAAACCACACAGACG	#23
*IL-36ϒ*	NM_000628.3	GGTCGTGTGCTTGGAGGA GGTACCATTCCCAATGCTGA	#20
*IL-38*	NM_018724.3	AAGAAGGACCTCCGGCTCT TGACTCAGAATCTGGC5GTATTTC	#69
*IL-36RN*	NM_014432.2	TCCATCAACATGAAGAATGTCC AGCCATTTCTTTTGCCCATA	#22
*GADPH*	NM_002046.3	AGCCACATCGCTCAGACAC GCCCAATACGACCAAATCC	#60

**Table 2:** Demographic and clinical characteristics of Crohn's disease and ulcerative colitis patients.

**Variable**	**Non-inflamed Controls (n=30)**	**Inactive UC patients (n=20)**	**Active UC patients (n=30)**	**Inactive CD patients (n=10)**	**Active CD patients (n=10)**
**Age, years**					
Mean	48.9	40.8	39.05	49.3	**25**
**Sex, **	14/16	6/14	10/20	4/6	3/7
**female/male**					
**Treatment**					
Mesalazine	*ND*	*19/20*	*19/30*	*1/10*	*1/10*
Azathioprine	*ND*	*4/20*	*1/30*	*5/10*	*3/10*
Prednisone	*ND*	*3/20*	*3/30*	*2/10*	*1/10*
Mercaptopurine	*ND*	*0/20*	*3/30*	*0/10*	*1/10*
**Disease **					
**Extension**	ND	7/20	1/30	1/10	0/10
Distal colitis	ND	12/20	8/30	7/10	6/10
Pancolitis					
**Extra-intestinal **					
**manifestations**					
absent	ND	15/20	17/30	7/10	6/10
present	ND	4/20	12/30	1/10	0/10

CD: Crohn's Disease patient group; UC: ulcerative colitis patient group. ND: not determined.

## Data Availability

The data used to support the findings of this study are available from the corresponding author upon request.
